# The association of asthma education centre characteristics on hospitalizations and emergency department visits in Ontario: a population-based study

**DOI:** 10.1186/s12913-014-0561-x

**Published:** 2014-11-13

**Authors:** Nancy J Garvey, Therese A Stukel, Jun Guan, Yan Lu, Phillip T Bwititi, Astrid Guttmann

**Affiliations:** Charles Sturt University, Wagga Wagga, New South Wales Australia; Institute for Clinical Evaluative Sciences, Toronto, Ontario Canada; Division of Paediatric Medicine, Hospital for Sick Children, Toronto, Ontario Canada; Department of Paediatrics University of Toronto, Toronto, Ontario Canada; Institute for Health Policy, Management and Evaluation, University of Toronto, Toronto, Ontario Canada

**Keywords:** Asthma, Asthma education, Hospitalizations, Emergency department visits, Outcomes

## Abstract

**Background:**

International guidelines recommend patient education as an essential component of optimal asthma management. Since 1990 hospital-based asthma education centres (AECs) have been established in Ontario, Canada. It is unknown whether patient outcomes are related to the level of services provided.

**Methods:**

Using linked, population-based health administrative and hospital survey data we analyzed a population of patients aged 2 to 55 years with a hospitalization for asthma (N = 12 029) or a high acuity asthma emergency department (ED) visit (N = 63 025) between April 2004 and March 2007 and followed for three years. Administrative data documenting individuals’ attendance at AECs were not available. Poisson models were used to test the association of potential access to various AEC service models (outpatient service availability and in-hospital services) with asthma readmissions, ED visits or death within 6 to 36 months following the index admission or ED visit.

**Results:**

Fifty three of 163 acute care hospitals had an AEC (N = 36) or had access by referral (N = 17). All AECs documented use with guideline-based recommendations for AE programs. ED patients having access to an AEC that offered full-time, extended hours had reduced rates of adverse outcomes (adjusted relative rate [aRR] 0.78, 95% confidence interval [CI] 0.69, 0.90) compared to those with no AEC access. Hospitalized patients with access to asthma education during hospitalization had reduced rates of adverse events (aRR 0.87, 95% CI 0.75, 1.00) compared to those with no inhospital AEC access.

**Conclusion:**

Although compliant with asthma guideline-based program elements, on a population basis access to asthma education centres is associated only with a modest benefit for some admitted and ED patients and depends on the level of access to services provided. Review of both services provided and strategies to address potential barriers to care are necessary.

## Background

Approximately 1.7 million of the 13 million children and adults in Ontario have been diagnosed with asthma, with the associated economic burden in 2011 estimated to be over $1.8 billion Canadian dollars [1]. Clinical practice guidelines for asthma recommend patient education as essential to optimize associated health outcomes and reduce healthcare costs [2-4]. To address this need in Ontario, Canada, over 30 hospital-based asthma education centres (AECs) were established between 1990 and 2004, funded through hospital global budgets and/or through industry contributions. AECs were tasked to integrate guideline-based patient education standards and specialized counseling skills into programs for adults, children and caregivers.

The Canadian Network for Respiratory Care originated in 1994 to address the growing need for specialized asthma education skills, knowledge and ability. A national certification program for the Certified Asthma Educator designation was established in 1999 [5], incorporating standards for guideline-based program content [2-4] and specialized training for staff. Key elements of asthma education included diagnosis, medications, inhaler devices, prevention of symptoms and attacks, signs of worsening asthma, monitoring asthma control, and need for medical attention [2]. Although clinical trials have shown asthma education to be effective in improving disease control [6-12], to our knowledge, there have not been any population-based studies on the effectiveness of multifaceted, hospital-based asthma education programs.

The aims of this study were to (i) describe the attributes of AECs in Ontario including education program and service availability characteristics, and (ii) assess whether patients hospitalized with asthma or having an emergency department (ED) visit for asthma with access to AECs had improved outcomes compared to those without access. We hypothesized that access to AECs with fulltime and extended hours for outpatient care, and asthma education during an admission or ED visit would be associated with reduced readmissions and ED visits.

## Methods

We undertook a retrospective population-based cohort study of patients with asthma seen in an acute care hospital or ED in Ontario, Canada between April 1, 2004 and March 31, 2007. Patients were followed for three years (through March 31, 2010). An AEC was a centre that provided a hospital-based program of asthma self-management education to children and/or adults with asthma.The research was approved by the research ethics boards at Charles Sturt University, protocol number 2007/103 and Sunnybrook Health Sciences Centre, Project Identification Number 353-2009.

### Data sources

#### Survey data

One hundred sixty three acute care hospitals in Ontario were contacted by phone to confirm AEC availability during 2004-2007. If the site was identified as potentially providing AEC service, an email including the study description, consent form and a link to an electronic survey was forwarded to the appropriate contact person. The survey was designed and beta tested based on a similar survey on diabetes patient education programs [13]. The survey identified onsite (primary) AECs and referral sites associated with the primary AEC, service delivery and guideline-based characteristics of the program.

AEC service characteristics included:

- Hours of operation: full-time (≥30 hrs./wk.), part-time (<30hr./wk.), regular (Monday through Friday 8AM to 4PM) or extended (before 8AM, after 4 PM and/or weekends) hours;
- AEC access at either a primary hospital site or by a pre-arranged referral relationship to a primary site; and
- Asthma education provided to inpatients and/or ED patients.

#### Health administrative data

Patient records were linked using unique, anonymized, encoded identifiers across multiple Ontario health administrative databases containing information on all publicly insured, medically necessary hospital and physician services. These included the Discharge Abstract Database for hospital admissions that includes the most responsible diagnosis for length of stay, secondary diagnosis codes, comorbidities present upon admission, and complications occurring during the hospital stay; the National Ambulatory Care Reporting System for ED visits; the Ontario Health Insurance Plan for physician billings that includes diagnosis codes; and the Registered Persons Database for patient demographic information and deaths. Neighborhood income was derived from Statistics Canada census estimates from 2006. Rurality was defined by patient postal code using the Rurality Index for Ontario (RIO) 2008 which accounts for population size and travel time [14].

### Study cohorts

We divided our cohort into those with a first (index) (i) admission to hospital or (ii) high acuity ED visit for asthma between April 1, 2004 and March 31, 2007. Asthma was determined using the Most Responsible Diagnosis and based on the International Classification of Disease 10^th^ revision-Canada diagnosis codes of J45 (asthma); or R05 (cough) or R06 (abnormalities of breathing) with a secondary diagnosis of J45. High acuity ED visits were determined using the Canadian Triage Assessment Score (CTAS) levels 1-3 representing resuscitation, emergent, and urgent, respectively.

We excluded children under 2 and adults over 55 years of age to reduce the likelihood of other diagnoses that would likely not be cared for in an AEC (bronchiolitis in the young and chronic obstructive pulmonary disease in the elderly), patients transferred from other EDs, and those who did not survive 6 months after the index event.

### Outcomes

**Figure 1:**
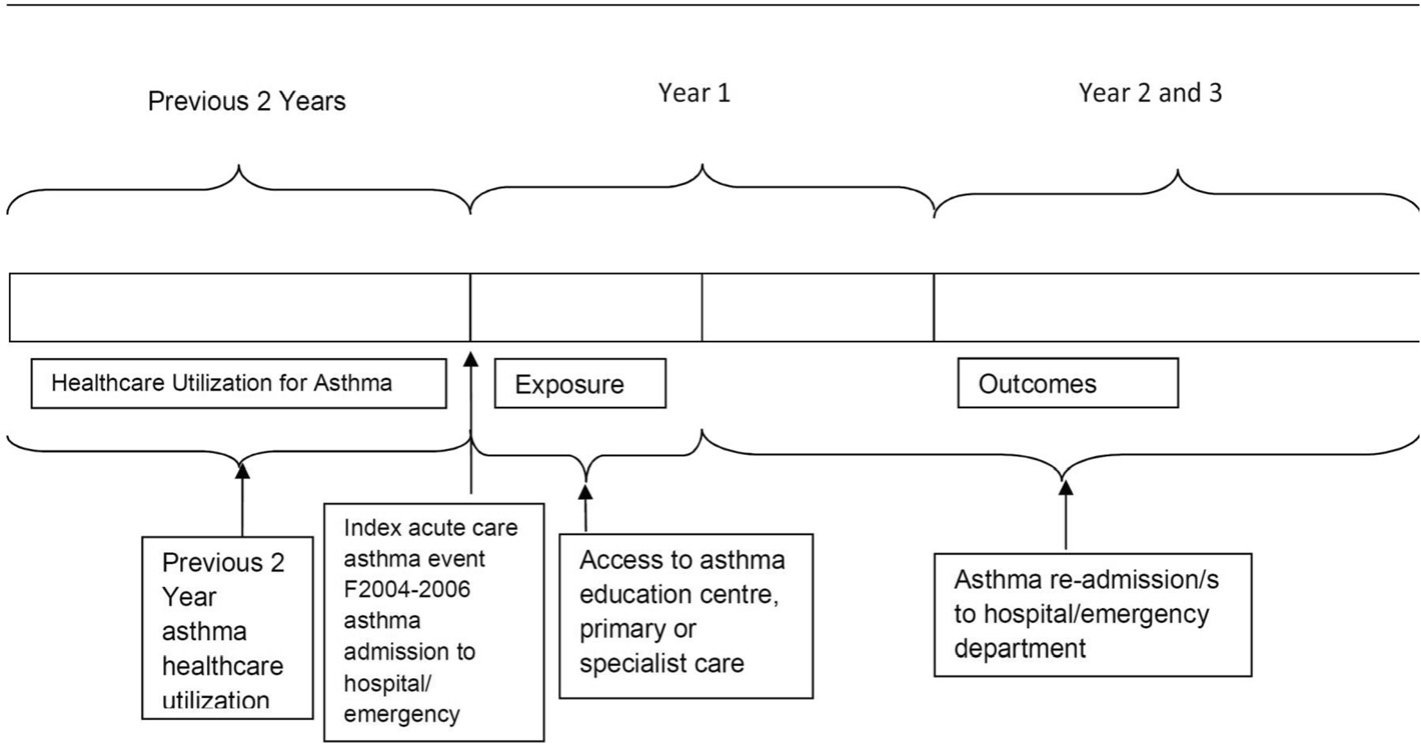
**AEC Study: Index Visit and Main Exposure Timeframes.**

The primary outcomes were readmission or high acuity ED visit for asthma or death from any cause during the 6 to 36 months after the index event to give patients a 6 month opportunity to access an AEC for follow up care (Figure 1). The primary exposure was access to an onsite or referral hospital-based AEC for outpatient follow up care at the time of the index event, categorized according to AEC service availability, defined as full-time or part-time, and having regular or extended hours of operation. The secondary exposure was asthma education availability to inpatients or ED patients. We could ascertain only whether the AEC service was available at the index hospital but not whether patients actually used the service.

Covariates included patient risk factors associated with asthma outcomes, including age [15], sex, [16] socioeconomic status [17], rural residence [18], history of asthma admissions or ED visits in the previous two years (as a measure of disease severity), and health services use in the two years prior to the index event including any primary care visit (as a proxy for access to primary care) and any specialist visit for asthma. We controlled for a history of asthma based on the Ontario Asthma Surveillance and Information System, a validated registry using administrative data to define incident and prevalent cases of asthma in Ontario [19]. We also controlled for hospital type: teaching, large community or small.

### Analysis

We used Poisson regression to analyze the association between AEC access and outcomes, controlling for patient risk and accounting for clustering within the initial hospital using generalized estimating equation models [20,21]. Patient risk factors included age, sex, asthma prevalence, history of primary care and specialist visits, previous ED visits and hospitalizations for asthma, neighbourhood income quintile, rural residence and hospital type.

Secondary analyses tested the association between availability of inpatient or ED asthma education during the index event and the outcomes. SAS v9.2.1 was used for analyses [22].

## Results

### Asthma education centres and survey data

#### Access to asthma education

Of the 163 acute care facilities contacted by phone, 43 hospital corporations were identified as potentially meeting the definition of an AEC. Five were excluded as they did not provide an organized asthma education program and 5 did not provide access to an AEC for greater than one year during the study period. Of the remaining 33, two did not reply.

Completed surveys from the other 31 hospital corporations documented the provision of hospital-based AEC services at 32 primary locations (one hospital site provided part-time primary services at two hospitals’ locations). The 32 primary hospital sites received referrals from 17 associated hospitals. The 2 hospital corporations that did not reply had publicly available information on their AEC services for four primary hospital sites. This information included hours of operation, programs for children and adults, and access to inpatient and emergency department asthma education interventions. The public information was confirmed by Nancy Garvey with the appropriate Respiratory Therapy Directors.

In summary, there were 53 hospital-based AEC sites that consisted of 36 primary sites and 17 referral sites providing referrals to primary sites. 110 hospitals had no access to AEC services.

#### Elements of asthma education programs

The survey revealed all of the responding sites were compliant with guideline-based program elements with minor variations in 2 of 16, namely they did not include peak flow monitoring or behavior modification techniques (Table 1).

**Table 1:** **Asthma education centre guideline-based program and service delivery characteristics for hospital-based AECs in Ontario**

	**Yes**
**Program characteristics**	N = 32*
**Population served**	
Adults	29 (91%)
Pediatrics/caregivers	30 (94%)
**Asthma education and resources provided in other languages**	7 (22%)
**Guideline-based program characteristics**	
Patient assessment	32 (100%)
Information provided about what asthma is	32 (100%)
Identification of individual risk/trigger Factors	32 (100%)
Review of asthma medications	32 (100%)
Difference between “relievers” and “controllers”	32 (100%)
Potential side effects of medications	32 (100%)
Review of asthma medication administration techniques	32 (100%)
Prevention of symptoms and attacks	32 (100%)
Signs that suggest asthma is worsening	32 (100%)
Coping strategies (e.g., how to deal with teachers/employers/healthcare professionals)	32 (100%)
Monitoring control of asthma	32 (100%)
Peak flow monitoring	28 (88%)
Environmental control	32 (100%)
How and when to seek medical attention	32 (100%)
Written action plan	32 (100%)
Behavior modification approach	29 (91%)
**Human resources**	
Certified Asthma Educators	28 (88%)
Other multi-disciplinary team members (Social Worker, Pharmacist)	5 (16%)
**Service delivery characteristics**	N = 36*
**Hours of service**	
Full-time regular hours (Monday through Friday 8AM to 4PM)	5 (14%)
Full-time regular and extended hours (before 8AM, after 4 PM and/or weekends)	10 (28%)
Part-time (less than 30 hrs. per week) regular hours	9 (25%)
Part-time regular and extended hours	12 (33%)
**Inpatient or ED asthma education provided by AEC staff**	
Inpatient education	22 (61%)
ED education	14 (39%)

*N = 32 primary sites with survey responses; N = 36 primary sites with service delivery information in survey and/or in the public domain.

### Study cohort

**Figure 2:**
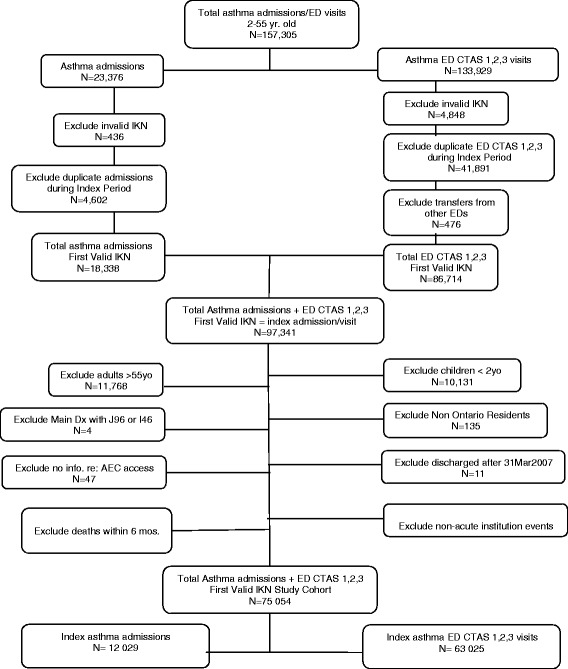
**Identification of Study Cohort April 1, 2004 through March 31, 2007.** *IKN = a unique anonymous Institute for Clinical Evaluative Sciences (ICES) key number

After applying all exclusion criteria (Figure 2), the final cohort consisted of 75 054 patients, 12 029 with an index admission and 63 025 with an index ED visit. Table 2 reports the baseline characteristics of the cohorts according to access to AEC service availability. Almost half (44%) did not have access to AECs. Patient characteristics differed across AEC service availability groups. Patients with more severe asthma, based on prior hospital admissions and high acuity CTAS scores, tended to be seen in hospitals with fulltime extended hours AECs.

**Table 2:** **Cohort baseline characteristics, according to AEC service availability**

	**Fulltime extended hours**	**Part-time extended hours**	**Fulltime regular hours**	**Part-time regular hours**	**No AEC access**
					**N = 33 353**
	**N = 9 857**	**N = 17 780**	**N = 6 365**	**N = 7 699**	
	**n (%)**	**n (%)**	**n (%)**	**n (%)**	**n (%)**
Male	5 052 (51.3%)	8 993 (50.6%)	3 044 (47.8%)	4 017 (52.2%)	16 491 (49.4%)
Age group (years)					
2-4	2 005 (20.3%)	3 329 (18.7%)	847 (13.3%)	1 956 (25.4%)	5 392 (16.2%)
5-9	1 671 (17.0%)	3 055 (17.2%)	784 (12.3%)	1 493 (19.4%)	5 165 (15.5%)
10-14	972 (9.9%)	1 762 (9.9%)	560 (8.8%)	794 (10.3%)	3 336 (10.0%)
15-19	738 (7.5%)	1 394 (7.8%)	585 (9.2%)	521 (6.8%)	2 907 (8.7%)
20-24	835 (8.5%)	1 534 (8.6%)	674 (10.6%)	541 (7.0%)	2 831 (8.5%)
25-29	710 (7.2%)	1 323 (7.4%)	587 (9.2%)	446 (5.8%)	2 494 (7.5%)
30-39	1 218 (12.4%)	2 257 (12.7%)	1 012 (15.9%)	865 (11.2%)	4 725 (14.2%)
40-55	1 708 (17.3%)	3 126 (17.6%)	1 316 (20.7%)	1 083 (14.1%)	6 503 (19.5%)
Income quintile					
1 (lowest)	2 244 (22.8%)	4 893 (27.5%)	1 735 (27.3%)	2 200 (28.6%)	7 826 (23.5%)
2	2 349 (23.8%)	4 047 (22.8%)	1 370 (21.5%)	1 599 (20.8%)	6 820 (20.4%)
3	2 383 (24.2%)	3 301 (18.6%)	1 220 (19.2%)	1 336 (17.4%)	6 466 (19.4%)
4	1 716 (17.4%)	2 999 (16.9%)	1 040 (16.3%)	1 265 (16.4%)	6 573 (19.7%)
5 (highest)	1 150 (11.7%)	2 511 (14.1%)	979 (15.4%)	1 256 (16.3%)	5 578 (16.7%)
Missing	15 (0.2%)	29 (0.2%)	21 (0.3%)	43 (0.6%)	90 (0.3%)
Rural	283 (2.9%)	519 (2.9%)	1 333 (20.9%)	502 (6.5%)	6 496 (19.5%)
Acuity at index event					
Inpatient admission					
	2 237 (22.7%)	3 060 (17.2%)	722 (11.3%)	1 145 (14.9%)	4 865 (14.6%)
Highest acuity CTAS 1-2 ED Visit					
	2 402 (24.4%)	3 971 (22.3%)	1 079 (17.0%)	1 530 (19.9%)	5 456 (16.4%)
Urgent acuity (CTAS 3) ED Visit					
	5 218 (52.9%)	10 749 (60.5%)	4 564 (71.7%)	5 024 (65.3%)	23 032 (69.1%)
Any primary care services in previous 2 years					
	9 232 (93.7%)	16 996 (95.6%)	5 851 (91.9%)	7 286 (94.6%)	31 535 (94.5%)
Pre-existing asthma					
	7 653 (77.6%)	13 819 (77.7%)	4 796 (75.3%)	5 774 (75.0%)	26 323 (78.9%)
Specialist asthma visit in previous 2 years					
	5 104 (51.8%)	9 814 (55.2%)	2 607 (41.0%)	4 539 (59.0%)	16 934 (50.8%)
Asthma admissions in previous 2 years					
	414 (4.2%)	641 (3.6%)	155 (2.4%)	286 (3.7%)	1 021 (3.1%)
Asthma ED visits in previous 2 years					
	1 390 (14.1%)	2 735 (15.4%)	952 (15.0%)	1 106 (14.4%)	4 906 (14.7%)
Hospital type					
Community	8 716 (88.4%)	13 744 (77.3%)	4 355 (68.4%)	5 888 (76.5%)	25 941 (77.8%)
Small	845 (8.6%)	0 (0%)	272 (4.3%)	0 (0%)	3 265 (9.8%)
Teaching	296 (3.0%)	4 036 (22.7%)	1 738 (27.3%)	1 811 (23.5%)	4 147 (12.4%)

### Outcomes

Overall, 8 230 (24%) of the ED cohort and 3 569 (50%) of the admission cohort had an asthma ED visit or admission in the 6-36 months following the index event. Table 3 describes the outcomes for each cohort according to AEC service availability. For both cohorts, the percentage of patients with no subsequent ED visits was highest in the AEC full-time/extended hours group.

**Table 3:** **ED visit, hospital admission or death within 6 months for the asthma admission and ED cohorts, according to AEC service availability**

	**Fulltime/extended hours**	**Part-time/extended hours**	**Fulltime regular hours**	**Part-time regular hours**	**No AEC**
**Admission cohort**					
	N = 2 237	N = 3 060	N = 722	N = 1 145	N = 4 865
ED visits for asthma within 6-36 months of index event					
0	1 535 (68.6%)	1 934 (63.2%)	441 (61.1%)	715 (62.4%)	3 131 (64.4%)
1	397 (17.7%)	580 (19.0%)	138 (19.1%)	216 (18.9%)	876 (18.0%)
2+	305 (13.6%)	546 (17.8%)	143 (19.8%)	214 (18.7%)	858 (17.6%)
Hospital admissions for asthma within 6-36 months of index event					
0	1 917 (85.7%)	2 624 (85.8%)	625 (86.6%)	968 (84.5%)	4 143 (85.2%)
1	232 (10.4%)	297 (9.7%)	61 (8.4%)	127 (11.1%)	489 (10.1%)
2+	88 (3.9%)	139 (4.5%)	36 (5.0%)	50 (4.4%)	233 (4.8%)
Any death between 6 and 36 months of index event					
Yes	12 (0.5%)	11 (0.4%)	*(<1%)	12 (1.0%)	43 (0.9%)
**ED cohort**					
	N = 7 620	N = 14 720	N = 5 643	N = 6 554	N = 28 488
ED visits for asthma within 6-36 months of index event					
0	6 008 (78.8%)	11 345 (77.1%) )	4 336 (76.8%)	5 005 (76.4%)	22 067 (77.5%)
1	1 072 (14.1%)	2 050 (13.9%)	804 (14.2%)	950 (14.5%)	4 042 (14.2%)
2+	540 (7.1%)	1 325 (9.0%)	503 (8.9%)	599 (9.1%)	2 379 (8.4%)
Hospital admissions for asthma within 6-36 months of index event					
0	7 534 (98.9%)	14 544 (98.8%)	5 599 (99.2%)	6 468 (98.7%)	28 176 (98.9%)
1	78 (1.0%)	159 (1.1%)	39 (0.7%)	76 (1.2%)	274 (1.0%)
2+	8 (0.1%)	17 (0.1%)	*	10 (0.2%)	38 (0.1%)
Any death between 6 and36 months of index event					
Yes	29 (0.4%)	73 (0.5%)	28 (0.5%)	36 (0.5%)	116 (0.4%)

*value suppressed due to small cell size.

Adjusting for covariates, there was a reduced rate of ED visits or hospitalizations for ED patients with access to AECs that offered full time extended hours as compared with those having no access to an AEC: adjusted relative rate (aRR), 0.78 (95% confidence interval (CI), 0.69 - 0.90) (Table 4). Access to asthma education services during the inpatient stay was associated with lower rates of admissions or ED visits for patients hospitalized with asthma (Table 5).

**Table 4:** **Adjusted relative rate (95% confidence interval (CI)) of asthma admission, ED visit or death within 6 to 36 months post index event for the asthma admission and ED cohorts, according to AEC service availability**

	**Admission cohort**	**ED cohort**
	**N = 12 029**	**N = 63 025**
	**Relative rate**	**Relative rate**
	**(95% CI)**	**( 95% CI )**
Access to AEC		
Fulltime extended hours	0.87 (0.71,1.08)	0.78 (0.69,0.90)
Fulltime regular hours	0.95 (0.74,1.22)	1.13 (0.98,1.30)
Part-time extended hours	0.90 (0.78,1.03)	0.94 (0.85,1.03)
Part-time regular hours	0.93 (0.80,1.08)	1.03 (0.84,1.25)
No AEC	1.00 Reference	1.00 Reference
Sex		
Male	1.04 (0.95,1.14)	1.14 (1.08,1.20)
Age group (years)		
2 - 4	0.57 (0.47,0.69)	0.93 (0.85,1.02)
5 - 9	0.51 (0.41,0.63)	0.81 (0.74.0.90)
10-14	0.48 (0.39,0.58)	0.69 (0.63,0.76)
15-19	0.87 (0.68,1.11)	0.93 (0.84,1.04)
20-24	1.00 Reference	1.00 Reference
25-29	0.78 (0.62,0.98)	0.91 (0.82,1.00)
30-39	0.95 (0.74,1.23)	0.89 (0.81,0.98)
40-55	0.66 (0.53,0.83)	0.86 (0.79,0.94)
Acuity at index event		
CTAS 1-2 (Highest acuity)	Not applicable	1.35 (1.27,1.43)
CTAS 3	Not applicable	1.00 Reference
Income quintile		
1 (lowest)	1.40 (1.23,1.59)	1.34 (1.25,1.44)
2	1.29 (1.13,1.46)	1.18 (1.10,1.27)
3	1.15 (1.01,1.31)	1.18 (1.10,1.27)
4	1.06 (0.94,1.20)	1.01 (0.94,1.07)
5 (highest)	1.00 Reference	1.00 Reference
Missing	1.72 (1.07,2.77)	0.92 (0.64,1.32)
Rural	1.01 (0.89,1.13)	0.99 (0.91,1.07)
Core primary care services in previous 2 yrs.	0.96 (0.78,1.18)	0.97 (0.88,1.07)
Previously diagnosed asthma	1.35 (1.21,1.50)	2.19 (2.01,2.38)
Specialist asthma visit in previous 2 yrs.	1.14 (1.07,1.22)	1.09 (1.04,1.14)
Asthma admissions in previous 2 yrs.	1.90 (1.67,2.16)	1.52 (1.35,1.72)
Asthma ED visits in previous 2 yrs.	2.47 (2.29,2.67)	2.71 (2.53,2.89)
Hospital type		
Community	1.01 (0.84,1.22)	1.26 (1.12,1.43)
Small	0.82 (0.62,1.08)	1.4 (1.18,1.66)
Teaching	1.00 Reference	1.00 Reference

**Table 5:** **Adjusted relative rate (95% confidence interval (CI)) of asthma admission, ED visit or death within 6 to 36 months post index event for the asthma admission and ED cohorts, according to access to inpatient or ED asthma education**

	Adjusted Relative Rate* (95% confidence intervals)
**ED cohort**	
Access to AE including in ED	0.91 (0.76,1.10)
Access to AEC but no AE in ED	0.97 (0.89, 1.06)
No AEC	1.00 Reference
**Admission cohort**	
Access to AE including during hospitalization	0.87 (0.75,1.00)
Access to AEC but no AE during hospitalization	0.95 (0.84, 1.07)
No AEC	1.00 Reference

*Adjusted for age, gender, socioeconomic status, rural residence, history of prior asthma admissions, primary and/or specialist asthma care, and hospital type**.**

## Discussion

Hospital-based AECs in Ontario have been implemented locally through hospital’s global budgets and/or industry-supported funding. However, in this large, population-based study, we found only a modest association between potential AEC access and reduced subsequent acute care use for asthma. ED patients with asthma with access to an AEC having fulltime and extended hours of service had lower rates of admissions and ED visits in the 6-36 months follow up period. Hospitalized asthma patients with access to inpatient asthma education had lower rates of readmission and ED visits. Almost half of patients with asthma had no AEC access at their hospital or ED.

The evidence for the benefits of asthma education exists in many studies [4-10,23-29]. Three Cochrane reviews associate educational interventions with lower risk of future ED visits and in some cases, hospital admissions for asthma along with other improvements in asthma-related health outcomes. A review of 32 single-centre studies of asthma in children by Wolf et al showed that asthma self-management education programs had modest reductions in emergency room visits, with a more pronounced effect for those having moderate or severe disease [6]. Boyd et al., in their review of 17 trials related to children who attended the emergency department for asthma, reported a lower risk of repeat visits and hospital admissions when children received an educational intervention [7]. In a review of 15 trials, Gibson et al reported that for adults, optimal education in asthma self-management resulted in improvements in asthma outcomes [8].

In Ontario, access to care is not an issue of health insurance. However, other barriers to access to chronic disease programs have been identified [30,31]. Recommendations to address these include the need for programs to be provided at a local level, improving ease of access and extended hours so that patients do not need to leave work for care, reducing travelling times, and tailoring programs to local needs. Our findings that only extended hours for AECs are associated with improved outcomes provides empiric support for the need for flexible services.

The socio-economic gradient in rate of readmissions and ED revisits in our cohort suggests that other barriers to care exist in a universal healthcare system. Others have demonstrated the relationship of poor asthma outcomes to medication insurance and access to controller medications [32], and environmental triggers in the home [33] or work environment [34].

Our study is the first to study the effectiveness of asthma education programs including AEC program and service characteristics in a “real world” setting. Although our survey indicates that AECs in Ontario incorporate guideline-based recommendations for asthma education into their programs and use trained Certified Asthma Educators as the primary healthcare providers for patient education, our findings in the context of the evidence for asthma education suggest that barriers to access to asthma education services are an important issue.

Asthma is considered to be an ambulatory care sensitive condition, one where appropriate ambulatory care may prevent or reduce the need for admission to hospital [35-39]. This study focused on the potential for multi-modal asthma education to prevent acute exacerbations after an admission or high acuity ED visit. However, preventing these initial events is also important. Two Ontario studies demonstrated significant reductions in ED visits and improved asthma-related health outcomes for those receiving asthma self-management education from Certified Asthma Educators in primary care [24,25]. Over 90% of patients in our study were seen by primary care providers in the previous two years. This suggests there is the potential to further reduce asthma morbidity, as measured by the index events in our study, with self-management programs integrated into primary care. This could increase access to asthma education for those without local access to an AEC.

In the secondary analysis of AEC services at the time of the index event, the finding of effectiveness of inpatient compared with ED asthma education is noteworthy. Our study could not determine what proportion of patients actually had asthma education during their index event. However, our findings suggest that there may be specific issues delivering effective asthma education in an ED setting, such as the relatively short timeframe that patients will spend in the ED as compared with inpatients. Research suggests in-hospital asthma care can provide a key teachable moment, when patients and their caregivers have a stronger motivation to learn [40].

### Strengths and limitations of the study

Strengths of this study include high survey response rates and population-based study of the effectiveness of AECs. The survey allowed definition of AEC services in a highly operational way that underscored differences in access to services while attesting to programs’ alignment with guideline-based recommendations for asthma self-management education.

The main limitations are related to our exposure of potential access to AECs since we were not able to ascertain which patients actually used these AEC services. Administrative data of attendance at AECs does not exist. In addition, patients identified as not having access could have attended an AEC at another institution. However, it was not our intent to evaluate asthma education at the individual patient level but to assess the potential benefit of these centres as currently implemented for their population of patients with acute asthma. It is also possible that hospitals that have AECs are different in other ways from those that do not. There may also be unmeasured confounding (patient or geographic). For instance, it is possible that some hospitals instituted AECs to address high regional rates of asthma or severe asthma due to local conditions such as high pollution. A higher proportion of patients with no AEC access live in rural areas, and there may be environmental differences that are related to disease severity or differing patterns of healthcare use that may explain our inconsistent findings around the benefit of AECs. We attempted to control for relevant patient and hospital characteristics but the observational nature of the study can point only to association and not causality.

## Conclusions

AECs in Ontario incorporate guideline-based recommendations for asthma education into their programs and use trained Certified Asthma Educators as the primary healthcare providers for patient education. Extended hours of service and inpatient asthma education appear to be a necessary component for effective care but overall effects on subsequent acute care use are modest. Our study suggests that current implementation and funding of AECs should include review of both the effectiveness of, and access to, these services. Administrative records of AEC attendance would facilitate monitoring and evaluation of these services.

### Prior abstract presentation

Canadian Respiratory Conference 2012, Vancouver BC, April 28, 2012.

American Thoracic Society International Conference, San Francisco CA, May 23, 2012.

INSPIRE 2012 Respiratory Therapy Society of Ontario Education Forum, Oakville ON, October 17, 2012.

Ontario Lung Association Better Breathing Annual Conference, Toronto ON, February 2, 2013.

## Abbreviations

aRR: Adjusted relative rate
AEC: Asthma education centre
CTAS: Canadian triage assessment system
ED: Emergency department
